# Repellent Activity of the Essential Oil from the Heartwood of *Pilgerodendron uviferum* (D. Don) Florin against *Aegorhinus superciliosus* (Coleoptera: Curculionidae)

**DOI:** 10.3390/molecules21040533

**Published:** 2016-04-22

**Authors:** Javier Espinoza, Alejandro Urzúa, Jocelyne Tampe, Leonardo Parra, Andrés Quiroz

**Affiliations:** 1 Laboratorio de Química Ecológica, Departamento de Ciencias del Ambiente, Universidad de Santiago de Chile, Casilla 40, Correo 33, Santiago 9170022, Chile; 2 Laboratorio de Ecología Química, Departamento de Ciencias Químicas y Recursos Naturales, Universidad de La Frontera, Casilla 54-D, Temuco 4811230, Chile; jositat@gmail.com (J.T.); leonardo.parra@ufrontera.cl (L.P.); andres.quiroz@ufrontera.cl (A.Q.)

**Keywords:** *Aegorhinus superciliosus*, *Pilgerodendron uviferum*, essential oil, sesquiterpenes, repellent effect

## Abstract

The weevil *Aegorhinus superciliosus* Guérin (Coleoptera: Curculionidae), which is endemic to Central-Southern Chile and Argentina, is one of the major berry pests in Chile and the most important pest in the La Araucanía Region (38°44′9″S, 72°35′25″W). Due to the poor effectiveness and problems surrounding the implementation of the traditional control methods using organophosphate and carbamate insecticides, new strategies for controlling this pest are needed. In this communication, we evaluated the behavioral responses of male and female *A. superciliosus* to volatile compounds released from the essential oil (EO) obtained from the heartwood of *Pilgerodendron uviferum* (D. Don) Florin using olfactometric bioassays. The composition of the EO was analyzed using gas chromatography (GC) and gas chromatography/mass spectrometry (GC/MS). According to these analyses, δ-cadinol (24.16%), cubenol (22.64%), 15-copaenol (15.46%) and δ-cadinene (10.81%) were the principal components of the EO. The *Pilgerodendron uviferum* EO, which is almost exclusively composed of sesquiterpenes (99.5%), exhibited a repellent effect against *A. superciliosus* adults, regardless of the sex or concentration used (56.6 mg/cm^3^ and 1.58 × 10^−2^ mg/cm^3^). The EO has low volatility and greater persistence than the EOs composed of monoterpenes and is considered a good model in the search for raspberry weevil repellents.

## 1. Introduction

*Aegorhinus superciliosus* Guérin (Coleoptera: Curculionidae), commonly known as the raspberry weevil, is endemic to Central-Southern Chile and Neuquén, Río Negro, Argentina. *A. superciliosus* is associated with deciduous forests and Valdivian temperate forests from the Maule to Los Lagos Regions (41°55′11″S, 72°8′29″W) [1] and is the most important pest in the La Araucanía Region because they feed on valuable berries including raspberries, blueberries and strawberries [2]. The larvae bore into the lateral roots with their powerful jaws, finally boring into the primary roots to build chambers to pupate, which may cause the death of the hosts. After pupal eclosion, the emerged adults cause damage to leaves, buds and fruits [3,4,5].

Only two parasitoids of *A. superciliosus* have been found in Chile*—Centistes* spp., a parasitoid wasp for the larvae, and a tachinid (Diptera), a parasitoid of adults—and they are unable to control the pest populations. Therefore, insecticide treatment is the main control strategy, followed to a lesser extent by biological, mechanical-physical and cultural control. Chemical control has limited effectiveness in adult insects, and it is ineffective against the larvae that populate the roots. Organophosphate and carbamate insecticides have been used to control this pest; however, these kind of compounds have negative effects on the environment [6,7], such as a long residual effect, exclusion period and toxicity to pollinators when applied close to the flowering period. The entomopathogenic fungi *Beauveria* and *Metarhizium* (Moniliales: Moniliaceae) have been used as biological treatments. These fungi are harmless to plants and animals [8], but so far, their use has not spread. Another control method investigated in recent years is the behavioral control of weevils using semiochemicals, reorted by Parra *et al*. [9] and Mutis *et al*. [5]. This promising line of work is currently being investigated.

The low efficiency and problems arising from the application of current control methods determined the urgent need for new strategies for controlling *A. superciliosus*. In this scenario, the use of natural products with properties that modify the behavior of *A. superciliosus*, such as essential oils (EOs), is viewed as an important alternative [6]. EOs have a wide spectrum of biological activities against insects: they can act as insecticides [10], repellents, antifeedants, growth regulators and oviposition deterrents [11]. Additionally, EOs and their constituents are not toxic and qualify as “low-risk pesticides” [6,12].

The examination of the literature on the repellent activity of EOs against Coleoptera species shows that the principal components of the active oils tend to be monoterpenoids, and little is known about the repellent activity of sesquiterpene-rich essential oils [13,14]. In this communication we report the behavioral response of *A. superciliosus* males and females to volatile compounds released from *Pilgerodendron uviferum* (D. Don) Florin (Cupressaceae) EO, which is almost exclusively composed of sesquiterpenes [15].

*Pilgerodendron uviferum* (D. Don) Florin (Cupressaceae) is a tree native to southern Chile and Argentina. Its wood is resistant to degradation, and it has been postulated that its resistance to microorganisms and insects is related to its chemical composition [15,16]. In this communication, we report the composition of the EO obtained by hydrodistillation from the fresh heartwood of *P. uviferum*, which was determined using gas chromatography (GC) and gas chromatography/mass spectrometry (GC/MS), and the behavioral responses of male and female *A. superciliosus* to the *P. uviferum* EO at two different concentrations using a four-arm olfactometer.

## 2. Results and Discussion

After fresh milling of *P. uviferum* heartwood (2.0 kg), 26.31 g of EO was obtained (1.4%). The EO was analyzed by GC/MS (Table 1). Twenty compounds were identified in the EO, corresponding to 87.05% of all detected compound. Three monoterpenes (0.43%)—two non-oxygenated (0.36%) and one oxygenated (0.06%)—and 17 sesquiterpenes (86.62%)—12 non-oxygenated and five oxygenated—were present in the EO. The sesquiterpenes δ-cadinene (10.81%), δ-cadinol (24.16%), cubenol (22.64%) and 15-copaenol (15.46%) were the most abundant compounds in the EO. Identification was corroborated by studying the fragmentation patterns in the obtained mass spectra. Although the relative abundance of the monoterpenes is low, this is the first report of the presence of monoterpenes in the EO from *P. uviferum* heartwood, highlighting the usefulness and advantages of GC/MS for studying volatile compounds compared to other extractives techniques that involved successive evaporations.

The sesquiterpene nature of the heartwood EO agrees with the report of Oyarzún and Garbarino [17], who isolated the sesquiterpenes (−)-*trans*-calamenene, (+)-δ-cadinene, (−)-caryophyllene-4,5-epoxide, (−)-humulene-1,2-epoxide, (−)-cubenol, (−)-*epi*-cubenol, (−)-torreyol and (−)-15-copaenol from the heartwood of *P. uviferum* collected in Chile. However, the composition of the EO from the heartwood of *P. uviferum* that we studied is very different from the composition of the EO from the leaves of *P. uviferum* collected in Argentina reported by Malizia *et al*. [18], which was rich in monoterpenes (54.1%), although with a large presence of sesquiterpenes (40.4%).

The olfactometric response of *A. superciliosus* adults toward the EO from *P. uviferum* was evaluated. The average time spent by individuals in the control zone was significantly higher (12.5 ± 1.3 min) than in the stimulus zone corresponding to diluted EO (1.58 × 10^−2^ mg/cm^3^) (6.9 ± 1.3 min) (*p* = 0.044) and was very significantly higher than in the decision zone (0.5 ± 0.1 min) (*p* < 0.0001). Compared with the pure EO (56.6 mg/cm^3^), the average time spent by individuals in the control zone was higher (12.1 ± 1.6 min) than in the stimulus zone (7.2 ± 1.6 min) and was significantly higher than in the decision zone (0.7 ± 0.4 min) (*p* < 0.0001). No differences were observed in the insect repellent response toward pure or diluted EO, although the dilute concentration was approximately 3500 times lower. Insects exposed to the pure EO sample decreased their movement and groomed their antennae continuously with their front legs. No signs of toxicity were observed. Therefore, the EO from the heartwood of *P. uviferum* repelled *A. superciliosus* adults.

**Figure 1:**
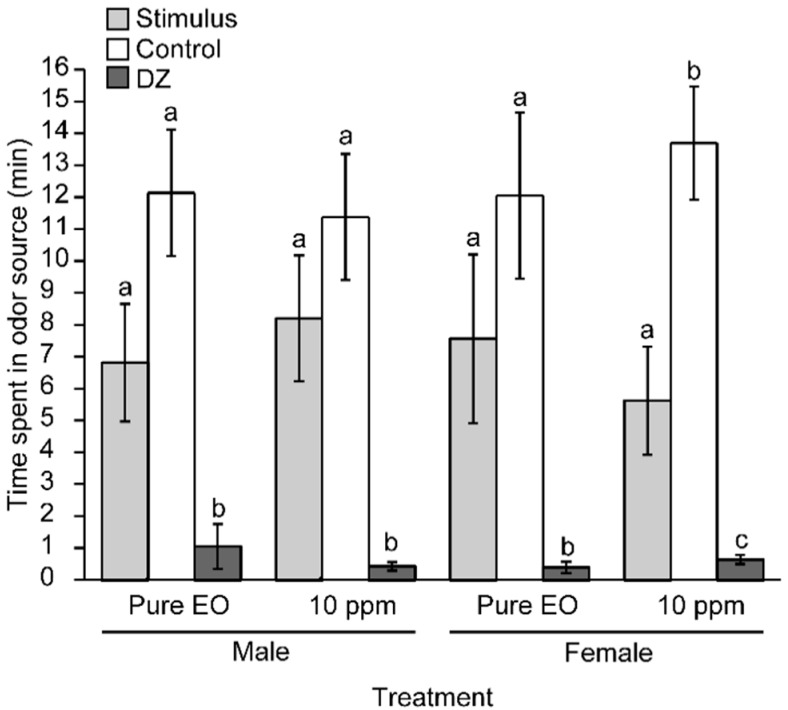
Average time spent (min ± SE) by *A. superciliosus* in the olfactometer arms containing pure EO and diluted *P. uviferum* heartwood EO (2 µL and 50 µL of 10 mg/L EO, *i.e.*, 56.6 mg/cm^3^ and 1.58 × 10^−2^ mg/cm^3^, respectively). Control: purified air, DZ: decision zone. The lines on the bars indicate the standard error. Different letters indicate significant differences based on the non-parametric Friedman test followed by the Conover-Inman test (*p* ≤ 0.05) N = 24.

Additionally, the olfactometric responses of male and female *A. superciliosus* toward the EO from the heartwood of *P. uviferum* were evaluated. The tendency of the insects to stay in the control zone longer than in the stimulus zone or decision zone remained (Figure 1). The insect responses were independent of sex and EO concentration (Figure 1). The EO from the heartwood of *P. uviferum* repelled both male and female *A. superciliosus*.

The behavior of *A. superciliosus* exposed to *P. uviferum* EO follows. In general, insects walked around the four arms of the olfactometer during the first half of the bioassay. Then, individuals chose and remained in one of the control arms until the end of the bioassay. No abdominal movements alluding to mating nor exposition of the *aedeagus* from male weevils were observed [19].

This is the first report of the behavioral response of male and female *A. superciliosus* to volatile compounds released from *P. uviferum* EO, which is almost exclusively composed of sesquiterpenes.

Among the few EOs rich in sesquiterpenoids that have been tested on Coleptera, we will mention the following: the EOs from the leaves and flowers of *Teucrium polium*, *Eupatorium inulaefolium* and *Eupatorium* *arnotti* that contain sesquiterpene-rich EOs show repellent and fumigant toxicity properties against the stored-product *Tribolium castaneum* [20,21]. Additionally, the EO from *Zanthoxylum dissitum* roots exhibited moderate contact toxicity against tree species of stored-product insects, including *Lasioderma serricorne* adults, *Tribolium castaneum* adults and *Attagenus piceus* larvae [22]*.* The EO from *Z. dissitum* leaves showed lower contact toxicity than the root EO. The major components in the EOs from *Z. dissitum* roots and leaves were humulene epoxide, caryophyllene oxide, δ-cadinol and caryophyllene. All of them are present in the EO from *P. uviferum*. The EO from *Chrysanthemum macrotum* caused significant mortality in larvae, adults and nymphs of *Tribolium confusum* by contact fumigant toxicity. The EO is rich in sesquiterpenes with cubenol, T-cadinol, α-cadinol and α-humulene being the major constituents. Cubenol is one of the major components in *P. uviferum* EO. Additionally, the EO from *Zanthoxylum dissitum* roots exhibited moderate contact toxicity against 3 species of storage pests, *i.e.*, *Lasioderma serricorne* adults*, Tribolium castaneum* adults and *Attagenus piceus* larvae, with LD_50_ values of 13.8, 43.7 and 96.8 μg/adult, respectively. The major components of *Z. dissitum* roots and leaves were humulene epoxide II (29.4%), caryophyllene oxide (24.0%), δ-cadinol (12.8%) and caryophyllene (12.7%) [22]. All of them are present in the EO of *P. uviferum*.

Our results are in agreement with the few reports on EOs, and some of the sesquiterpenes present in *P. uviferum* EO are also present in EOs that shown repellency and toxicity toward Coleoptera. Essential oils containing sesquiterpenes are less volatile than EOs containing monoterpenes because their constituents have lower vapor pressures [23,24]. Sesquiterpenes have average boiling points 100 °C higher than monoterpenes [25]. The resulting longer action and lower associated cost is promising for their application as biocides [26]. In addition, insects have difficulty building up resistance because EOs are complex mixtures of many compounds [27,28].

## 3. Experimental Section

### 3.1. Insects

The colonies of *A. superciliosus* used in this study were hand collected in the same phenological state at an experimental blueberry plantation in Collipulli, La Araucanía Region (37°57′29″S, 72°25′55″W), Chile, in November 2014. The individual weevils were transferred to a small cage and then reared in entomological cages (30 cm × 30 cm × 30 cm) at 26 ± 1 °C under a 16:8 light:dark cycle and 70% humidity. The adults were provided with fresh blueberry twigs and leaves.

### 3.2. Plant Material

*P. uviferum* heartwood was collected in February 2012 in the south of the Baker River Basin (47°1′23″S, 72°49′46″W), Aysén Region, Chile. Voucher specimens were deposited at Laboratorio de Química de la Madera, Facultad de Ciencias Forestales y de la Conservación de la Naturaleza, Universidad de Chile. The samples were identified by forestry engineer René Carmona.

### 3.3. Essential Oil Extraction and Analysis by GC/MS

The EO was extracted from 2 kg of milled *P. uviferum* heartwood by hydrodistillation for 6 h in a Clevenger-type apparatus. The EO was dried over anhydrous sodium sulfate. Analysis of the EO components was performed by gas chromatography and gas chromatography/mass spectrometry (GC/MS) using the instrumentation described below. Qualitative analysis was performed using a Thermo Scientific Trace GC Ultra linked to an ISQ quadrupole mass spectrometric detector with an integrated data system (Xcalibur 2.0, Thermo Fisher Scientific Inc., Waltham, MA, USA); quantitative analysis was carried out using a Shimadzu GC-9A gas chromatograph fitted with an FID-9 detector (Shimadzu Corporation, Kyoto, Japan). The same capillary column (Rtx-5 MS, film thickness 0.25 μm, 60 m × 0.25 mm, Restek Corporation, Bellefonte, PA, USA) was used in both instruments. The operating conditions were as follows: On-column injection; injector temperature, 250 °C; detector temperature, 280 °C; carrier gas, He at 1.25 mL/min; oven temperature program: 40 °C increase to 260 °C at 4 °C/min, and then 260 °C for 5 min. The mass spectra were obtained at an ionization voltage of 70 eV. Recording conditions employed a scan time of 1.5 s and a mass range of 40 to 400 amu. The identification of compounds in the chromatographic profiles was achieved by comparison of their mass spectra with a library database (NIST08, NIST, Gaithersburg, MD, USA) and by comparison of their calculated retention indices with those reported in the literature [29] for the same type of column.

### 3.4. Olfactometer Bioassays

The behavioral responses of male and female *A. superciliosus* to the *P. uviferum* EO at two different concentrations were tested using a four-arm olfactometer [9,30]. The olfactometer consists of three acrylic plates held together with four metal screws; the top plate has a hole in the center to introduce the insect, which is subsequently connected to a vacuum pump adapter. One hole in each of the four arms is connected to a glass tube containing filter paper impregnated with the odor stimulus or the control (See Supplementary Figure S1). The olfactometric assay was conducted according to the procedure of Tapia *et al*. [31], which consists of observing the movements of the insect in an area called the arena. The arena was divided into five zones: a central square zone called the decision zone and four zones corresponding to the olfactometer arms. Two of the arms were enriched with air containing the volatile EO as the stimuli, and as control zones, the other two arms contained dichloromethane (HPLC grade, Sigma Aldrich, Steinheim, Germany) if the EO sample was diluted or purified air if the EO sample was pure. The samples were prepared by applying either 2 μL of pure *P. uviferum* EO or 50 μL of the EO diluted in dichloromethane (10 mg/L) on Whatman no. 1 filter paper (0.5 cm wide by 3.5 cm long). The filter papers, which were impregnated with stimuli, were placed in glass tubes (7 cm long; 1.5 cm i.d.) in different arms of the olfactometer. The insects were placed in the center of the olfactometer, and a purified air flow (800 mL/min) was generated to carry the volatile stimuli from the arms to a central hole in the olfactometer. The air was purified through a column of activated carbon. The bioassay was performed during the morning, according to the observed period of more activity of *A. superciliosus*. The olfactometric response of each insect to the stimulus was registered for 20 min, and the time spent in each arm was processed using EthoVision 3.1 software [32]. Each treatment was replicated 24 times for each sex, first with the males and then with the females. A different individual was used in each replicate of the experiments and then was discarded. After each replicate the olfactometer was cleaned with dichloromethane, and dried for 20 min at 55 °C in a forced-air oven.

### 3.5. Data Analyses

The times spent by *A. superciliosus* in the stimulus and control zones of the olfactometer were compared through the non-parametric Friedman test (*p* ≤ 0.05) followed by the Conover-Inman test (*p* ≤ 0.05) using StatsDirect 3.1 [33] statistics software.

## Figures and Tables

**Table 1:** Composition of the essential oil from *P. uviferum* heartwood.

RT	RI	Compound	%	Identification
19.64	1033	*p*-Cymene	0.12	RI, MS
19.78	1037	Limonene	0.24	RI, MS
19.92	1042	Eucalyptol	0.06	RI, MS
29.54	1365	α-Cubebene	0.05	RI, MS
30.34	1394	Copaene	0.71	RI, MS
31.60	1444	Caryophyllene	1.27	RI, MS
31.93	1458	α-Guaiene	0.06	RI, MS
32.47	1479	Humulene	1.33	RI, MS
32.86	1494	β-Cadinene	1.37	RI, MS
33.49	1520	α-Amorphene	0.96	RI, MS
34.04	1544	δ-Cadinene	10.81	RI, MS
34.32	1556	1,4-Cadinadiene	0.31	RI, MS
34.46	1561	4,5,9,10-Dehydroisolongifolene	0.26	RI, MS
34.62	1568	α-Calacorene	0.95	RI, MS
35.56	1607	γ-Elemene	4.75	RI, MS
35.72	1615	Caryophyllene oxide	1.02	RI, MS
36.33	1642	1,2-Epoxyhumulene	0.51	RI, MS
36.65	1656	δ-Cadinol	24.16	RI, MS
36.99	1671	Cubenol	22.64	RI, MS
37.16	1678	15-Copaenol	15.46	RI, MS

RT: Retention time (min), RI: Retention index, %: Considering identified compounds only.

## Supplementary Materials

Supplementary materials can be accessed at: http://www.mdpi.com/1420-3049/21/4/533/s1.
