# Supplementary material for: Repellent Activity of the Essential Oil from the Heartwood of Pilgerodendron uviferum (D. Don) Florin against Aegorhinus superciliosus (Coleoptera: Curculionidae)
Source: Molecules. 2016 Apr 22;21(4):533. doi: 10.3390/molecules21040533 (PMC6274372; doi:10.3390/molecules21040533)
Supplement: Supplementary file 1 [file molecules-21-00533-s001.pdf]

Supplementary Materials: Repellent Activity of the Essential Oil from the Heartwood of *Pilgerodendron uviferum* (D. Don) Florin against *Aegorhinus superciliosus* (Coleoptera: Curculionidae)

Javier Espinoza, Alejandro Urzúa, Jocelyne Tampe, Leonardo Parra and Andrés Quiroz

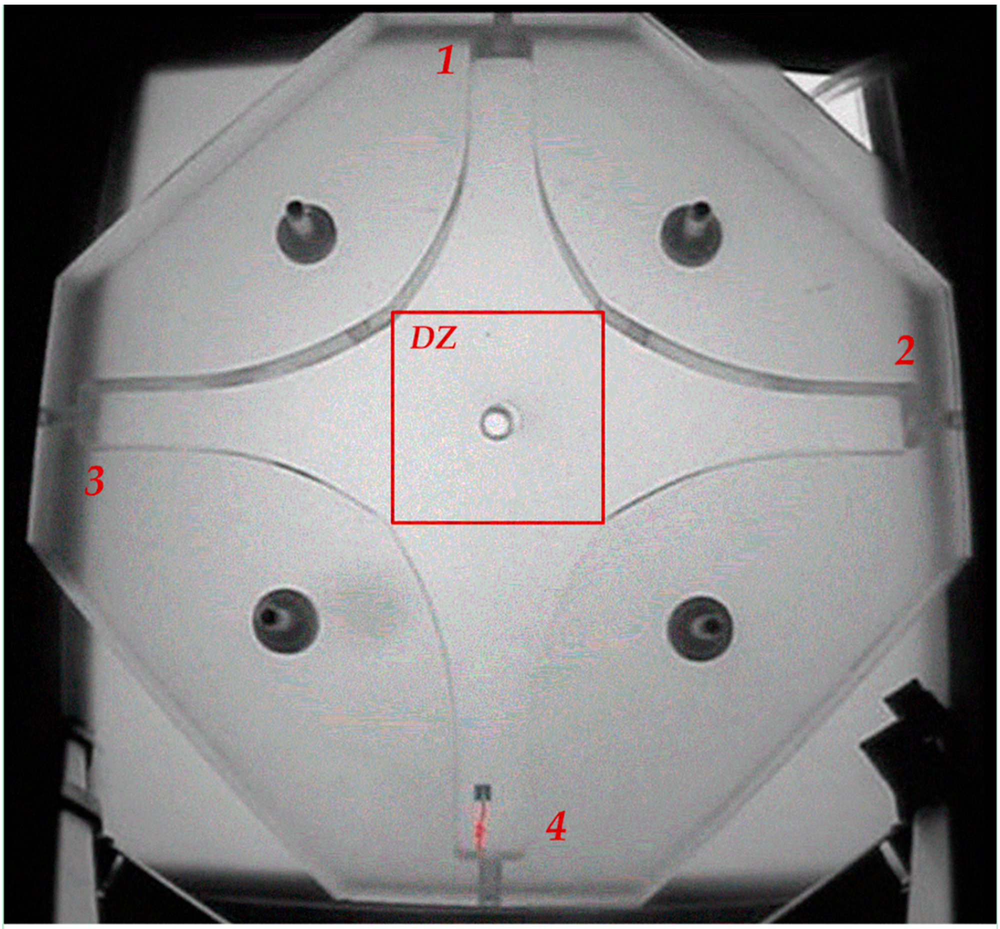

Figure S1. Four-arm olfactometer. 1 and 4 arms: Stimuli. 2 and 3 arms: Control. DZ: Decision zone.
